# Supplementary material for: A Simulation Study on the Processes of Intra-Group Informal Interaction Affecting Workers’ Safety Behaviors
Source: Int J Environ Res Public Health. 2022 Aug 15;19(16):10048. doi: 10.3390/ijerph191610048 (PMC9408364; doi:10.3390/ijerph191610048)
Supplement: Supplementary file 1 [file ijerph-19-10048-s001.zip › ijerph-1841937-supplementary.pdf]

Supplementary materials

This supplementary materials were utilized to briefly describe the process of questionnaire data analysis. The file includes two parts: concept measure scale, analysis tools and the results.

1. The measurement scales of related concepts

This study involves three four key concepts (variables), i.e., intra-group informal interaction (IGII), group knowledge sharing (GKS), group identification (GI), and workers' safety behaviors (WSB). The measurement scales of these concepts are presented in Table S1.

Table S1. Measurement scales of IGII, GKS, GI, and WSB.

● Measurement scale of IGII						
No.	Measurement question items	Strongly agree	Quite agree	Generally agree	Not quite agree	Disagree
1	The foreman and coworkers concern my real problems and needs	□	□	□	□	□
2	I and my coworkers help each other, we share our opinions and respect each other's views	□	□	□	□	□
3	I support the foreman when others have different opinions from him	□	□	□	□	□
4	I often have dinner or activities with the foreman and co-workers to share ideas and insights about life	□	□	□	□	□
5	I will sacrifice my interests to serve the foreman's and coworkers' interest	□	□	□	□	□
6	During the holidays, the foreman and I will visit each other and give each other holiday gifts	□	□	□	□	□
● Measurement scale of GKS						
1	I often share work reports and project department materials with group members	□	□	□	□	□
2	I will share my own safety work experience with group members	□	□	□	□	□
3	I often seek safety experiences from older members	□	□	□	□	□
4	The foreman and older members of the crew will share safety expertise and special techniques	□	□	□	□	□
● Measurement scale of GI						
1	Feeling an emotional attachment to our group	□	□	□	□	□
2	Feeling a strong sense of belonging to our group	□	□	□	□	□

3	Feel that the problems of the group are our problems	□	□	□	□	□
4	I feel like I am at home on the group	□	□	□	□	□
5	It makes sense for the foreman or seniors to ask me to do something	□	□	□	□	□
● Measurement scale of WSB						
1	I wear protective gear, such as helmets and face shields, in strict accordance with project safety regulations	□	□	□	□	□
2	I strictly follow safety management regulations and manuals for construction work	□	□	□	□	□
3	I conduct rigorous inspections of safety equipment before work	□	□	□	□	□
4	I will help my workers to ensure they work safely	□	□	□	□	□
5	I will actively participate in project safety education and training	□	□	□	□	□
6	I will actively do more work to ensure site safety	□	□	□	□	□

2. Data analysis tools and results

By using aforementioned scales, we conducted a questionnaire. The questionnaire was conducted for three months, a total of 278 questionnaires were collected, of which 271 were valid.

The remained data were import to SPSS 23 to conduct a correlation analysis. The correlation coefficient of these aforementioned concepts (i.e., IGII, GKS, GI, and WSB) were standardized and presented in the Figure S1.

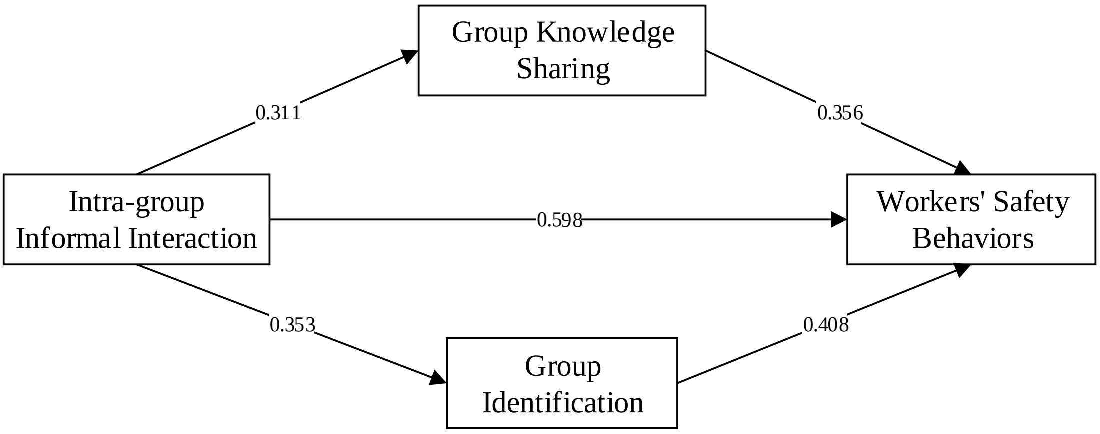

Figure S1. Correlations of IGII, GKS, GI, and WSB.
